# Mdivi-1, a mitochondrial fission inhibitor, modulates T helper cells and suppresses the development of experimental autoimmune encephalomyelitis

**DOI:** 10.1186/s12974-019-1542-0

**Published:** 2019-07-19

**Authors:** Yan-Hua Li, Fang Xu, Rodolfo Thome, Min-Fang Guo, Man-Luan Sun, Guo-Bin Song, Rui-lan Li, Zhi Chai, Bogoljub Ciric, A. M. Rostami, Mark Curtis, Cun-Gen Ma, Guang-Xian Zhang

**Affiliations:** 1 Department of Neurology, Thomas Jefferson University, Philadelphia, PA 19107 USA; 2 Institute of Brain Science, Shanxi Key Laboratory of Inflammatory Neurodegenerative Diseases, Shanxi Datong University, Datong, 037009 China; 3 “2011” Collaborative Innovation Center/Research Center of Neurobiology, Shanxi University of Traditional Chinese Medicine, Taiyuan, 030024 China; 4 Department of Pathology, Thomas Jefferson University, Philadelphia, PA 19107 USA

**Keywords:** Experimental autoimmune encephalomyelitis, Dynamin-related protein 1, Mdivi-1, T cells

## Abstract

**Background:**

Unrestrained activation of Th1 and Th17 cells is associated with the pathogenesis of multiple sclerosis and its animal model, experimental autoimmune encephalomyelitis (EAE). While inactivation of dynamin-related protein 1 (Drp1), a GTPase that regulates mitochondrial fission, can reduce EAE severity by protecting myelin from demyelination, its effect on immune responses in EAE has not yet been studied.

**Methods:**

We investigated the effect of Mdivi-1, a small molecule inhibitor of Drp1, on EAE. Clinical scores, inflammation, demyelination and Drp1 activation in the central nervous system (CNS), and T cell responses in both CNS and periphery were determined.

**Results:**

Mdivi-1 effectively suppressed EAE severity by reducing demyelination and cellular infiltration in the CNS. Mdivi-1 treatment decreased the phosphorylation of Drp1 (ser616) on CD4^+^ T cells, reduced the numbers of Th1 and Th17 cells, and increased Foxp3^+^ regulatory T cells in the CNS. Moreover, Mdivi-1 treatment effectively inhibited IFN-γ^+^, IL-17^+^, and GM-CSF^+^ CD4^+^ T cells, while it induced CD4^+^ Foxp3^+^ regulatory T cells in splenocytes by flow cytometry.

**Conclusions:**

Together, our results demonstrate that Mdivi-1 has therapeutic potential in EAE by modulating the balance between Th1/Th17 and regulatory T cells.

## Introduction

Multiple sclerosis (MS) is an immune-mediated chronic demyelinating disease of the central nervous system (CNS), characterized by inflammatory infiltrates, myelin loss, and axonal damage [17, 23]. Experimental autoimmune encephalomyelitis (EAE) is an animal model of MS that is often used to study the pathogenesis and therapeutics of the disease. EAE develops as myelin-specific CD4^+^ T cells penetrate into the CNS and secrete cytokines and other inflammatory mediators, which, in turn, recruit peripheral leukocytes that promote damage to the myelin sheaths [27].

In the pathogenesis of MS, two mutually complementary processes can be identified: autoimmune inflammation is more pronounced at the initial stages of the disease, while axonal neurodegeneration is observed in advanced disease [11]. Given that mitochondrial dysfunction is a major factor in the neurodegenerative process [20], targeting mitochondrial functions may be a promising therapeutic approach [12, 32]. Indeed, treatment with P110, a peptide inhibitor that targets dynamin-related protein 1 (Drp1), protects myelin in cuprizone-induced demyelination and EAE models by blocking Drp1 localization to the mitochondria, thus reducing mitochondrial fragmentation, oligodendroglial cell death, and microglia activation [16]. Mitochondrial division inhibitor-1 (Mdivi-1), similar to P110, is a highly efficient small molecule that selectively inhibits Drp1 self-assembly and GTPase activity, thus protecting target cells from oxidative stress and caspase-dependent and -independent apoptosis [10, 12, 26, 33, 35]. Mdivi-1 can cross the blood-brain barrier (BBB) and has a half-life of 12 h [5, 12]. Importantly, small molecule inhibitors are safer and less costly than peptide inhibitors. However, the effect of Mdivi-1 on immune responses in EAE remains to be determined.

In this study, we demonstrate that Mdivi-1 markedly inhibited Drp1 activation in CD4^+^ T cells, and suppressed EAE by modulating the infiltration of Th1 and Th17 cells while increasing the proportion of Foxp3^+^ Treg cells in the CNS. Mdivi-1 treatment also inhibited the expression of proinflammatory cytokines and upregulated the numbers of FoxP3^+^ Tregs in the periphery. Overall, our data show that Mdivi-1 has therapeutic potential in CNS autoimmune inflammation by modulating the immune response.

## Materials and methods

### Mice

Female C57BL/6 mice, 8–10 weeks of age, were purchased directly from the Jackson Laboratory (Bar Harbor, ME, USA). Experimental protocols were guided by the Institutional Animal Care and Use Committees of Thomas Jefferson University and Shanxi Datong University. All mice were bred in pathogen-free cages, under constant temperature, with free access to food throughout the experimental process.

### Induction and clinical evaluation of EAE

EAE was induced by subcutaneously immunizing mice with 200 μL of an emulsion containing 200 μg of myelin oligodendrocyte glycoprotein peptide 35–55 (MOG_35–55_ peptide, MEVGWYRSPFSRVVHLYRNGK, Genscript, NJ, USA) and an equal volume of Freund’s Complete adjuvant (Sigma-Aldrich, St. Louis, MO, USA) supplemented with 10 mg/mL of heat-killed *Mycobacterium tuberculosis* H37Ra (Difco, Detroit, MI, USA). Mice also received 200 ng of pertussis toxin (List Biologic Laboratories, Inc., Campbell, CA, USA) on days 0 and 2 post immunization (p.i.). Onset and progression of EAE signs were monitored daily on a 0–5 scale, where 0 = healthy, 1 = limp tail, 2 = hind limb weakness, 3 = paralysis of hind limb, 4 = tetraparalysis, and 5 = moribund/death. Finally, for each animal, we determined the time to disease onset, incidence rate, peak score, and cumulative disease score (sum of daily scores from disease onset to day 28).

### Mdivi-1 treatment

Mice were randomly divided into two groups: Mdivi-1-treated and DMSO-treated as control (*n* = 15 each group). Mdivi-1 was dissolved in dimethyl sulfoxide (0.1% DMSO). Mdivi-1 (25 mg/kg) or 0.1% DMSO was given intraperitoneally from day 3 p.i., until day 27 p.i.

### Histology and immunohistochemistry

In the mouse EAE model, the accumulation of autoreactive T cells was much greater in the dorsal blood vessels of the fifth lumbar spinal cord than in the ventral ones, suggesting dorsal blood vessels of the fifth spinal cord may be the gate for autoreactive T cells to enter the CNS [1, 15]. Lumbar spinal cords were therefore harvested after mice had been extensively perfused with saline and 4% PFA, and then fixed for 4 h in 2% PFA prior to OCT or paraffin embedding for pathological assessment. Consecutive 4 μm or 10 μm sections were taken, and Luxol Fast Blue (LFB) staining and hematoxylin and eosin (H&E) stained slices were analyzed in a blind fashion to assess basic histopathological changes. For immunohistochemistry [13], sections were preincubated for 2 h with the blocking buffer (1% BSA in PBS) and then incubated with one of the following polyclonal and monoclonal primary antibodies: anti-myelin basic protein (Millipore, Billericay, MA), anti-CD4, anti-IFN-γ, anti-IL-17 (BD, NJ, USA), anti-IL-10, and anti-pDrp1 (ser616) (Abcam, Cambridge, UK). As a negative control, additional sections were treated similarly, but the primary antibodies were omitted. Results were observed under confocal laser scanning microscope (CLSM, Olympus, Tokyo, Japan) using FV1200 software in a blinded fashion. Image-Pro Plus 6 was used to quantify staining areas and the number of infiltrating cells. Histological analyses were performed on at least two sections per mouse, and nine mice per group were analyzed.

### Preparation of mononuclear cells from spleen and the CNS

Mice were euthanized and perfused with cold saline at day 21 or 28 p.i. Spleens, brains, and spinal cords were collected in Iscove’s modified Dulbecco’s medium (IMDM, Thermo Fisher Scientific, Carlsbad, CA) supplemented with 10% heat-inactivated fetal bovine serum (Thermo Fisher Scientific), 100 U penicillin, 10 μg/mL streptomycin, 0.3 mg/mL l-glutamine, and 55 μM 2-mercaptoethanol. Single mononuclear cell (MNC) suspensions were prepared as described [28]. Spleens were mechanically disrupted through a 70-μm cell strainer, treated with RBC lysis buffer (Biolegend, CA, USA) for 1 min, and washed in IMDM. Brains and spinal cords were cut into pieces with scissors and enzymatically digested with 700 μg/mL Liberase TL (Roche, Nutley, NJ) for 30 min at 37 °C, filtered with a 70-μm cell strainer, and washed with IMDM. CNS MNCs were enriched by centrifuging cells in a 70/30% percoll gradient (Sigma-Aldrich, USA) for 20 min at 450 g.

### Flow cytometry analysis

One million MNCs were moved to FACS tubes, stimulated with PMA (50 ng/mL) and ionomycin (500 ng/mL) in the presence of GolgiPlug (1 μg/10^6^ cells, Sigma-Aldrich) for 4 h at 37 °C. For surface-maker staining, cells were incubated with fluorochrome-labeled Abs to CD4 (Clone GK1.5, BD Biosciences) in a final volume of 100 μL PBS at 4 °C for 20 min. For intracellular staining, cells were washed, fixed, and permeabilized using Fix and Perm cell permeabilization reagents (eBioscience). Intracellular cytokines were stained with Abs against IL-10 (Clone JES5-16E3, BD Biosciences), IL-17 (Clone TC11-18H10.1, Biolegend), IFN-γ (XMG 1.2, BD Biosciences), or Foxp3 (Clone FJK-16 s, eBioscience) in a final volume of 100 μL permeable solution for overnight at 4 °C. Samples were acquired on a FACSAria (BD Biosciences, USA). Instrument setup and calibration were performed daily using Cytometer Setup and Tracking beads (BD Biosciences, USA). In addition, cytometer configuration and compensation were set according to BD Biosciences recommendations. Data were analyzed with FlowJo Software (Treestar, Ashland, OR).

### Cytokine measurement by ELISA

Splenocytes were cultivated at a density of 1.0 × 10^6^ cells/mL in complete IMDM with or without 25 μg/mL MOG_35–55_ for 72 h. After the incubation period, supernatants were collected and assayed for IL-4, IL-5, IL-17, IFN-γ, GM-CSF, and IL-10 using ELISA kit (R&D System, Minneapolis, MN, USA). Determinations were performed in duplicate and results were expressed as pg/mL.

### Statistical analysis

Graphad Prism software (Cabit Information Technology, Shanghai, China) was used for statistical analysis. Experimental data were shown as mean ± SEM. Clinical mean score was analyzed by non-parametric Kruskal-Wallis test to determine whether an overall statistically significant change existed before using Mann-Whitney *U* test to analyze the difference between any two groups. Comparisons between two groups were carried out with Student’s *t* test. When comparing multiple groups, data were analyzed with one-way ANOVA with Tukey’s multiple comparisons test. A criterion of *P* < 0.05 was considered to be significant.

## Results

### Mdivi-1 alleviated the severity of EAE

**Fig. 1:**
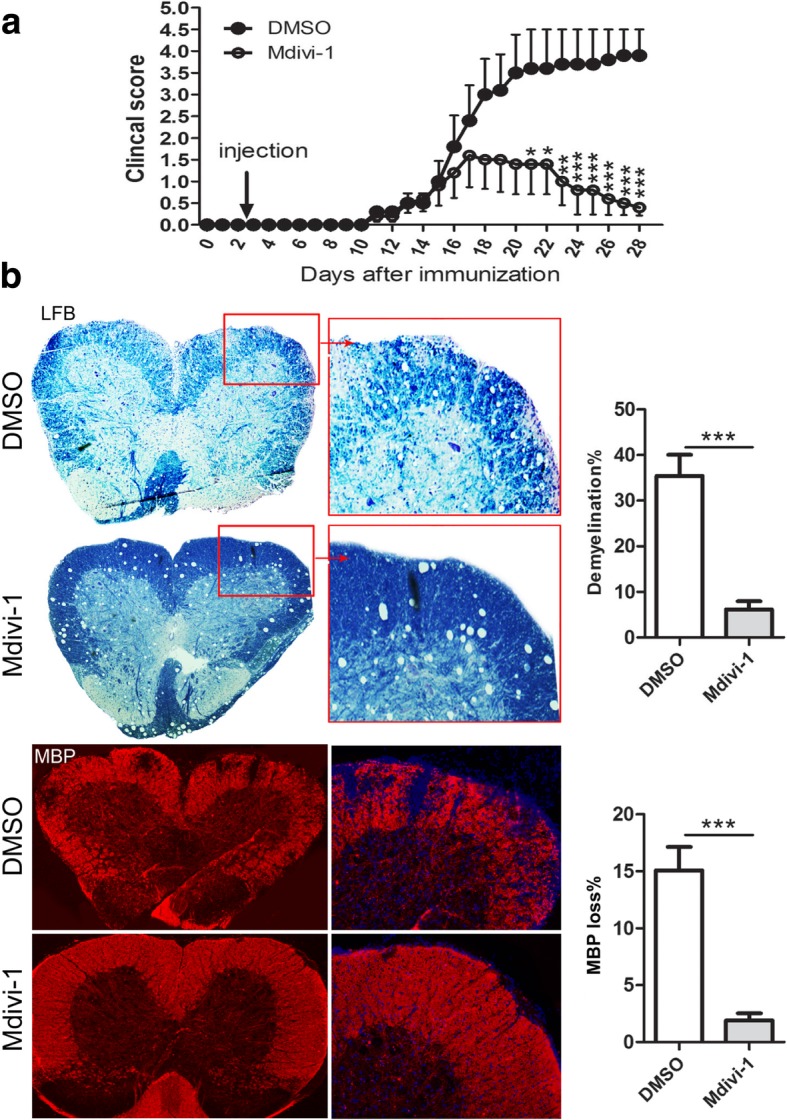
Mdivi-1 ameliorates the severity of EAE and demyelination. C57BL/6 mice were immunized with MOG_35–55_, Mdivi-1 (25 mg/kg) was given intraperitoneally, and 0.1% DMSO was established as control (*n* = 15 each group) in a similar manner on day 3 p.i., until day 27 p.i. Lumbar regions of spinal cords were harvested for LFB staining and immunostaining of MBP. **a** Clinical score of EAE mice, and **b** representative microphotographs for demyelination (Luxol Fast Blue staining and MBP immunostaining) and quantitative analysis of demyelination of whole spinal cord white matter lesions. Data represent mean ± SEM (*n* = 7–9 each group). *P < 0.05; **P < 0.01; ***P < 0.001. One representative of two experiments is shown

To investigate if Mdivi-1 suppresses EAE, mice were immunized with MOG_35–55_ peptide and treated with daily doses of Mdivi-1 (25 mg/kg) or vehicle (DMSO 0.1%) from day 3 p.i. until day 27 p.i. Our results show that EAE developed in DMSO and Mdivi-1-treated mice at day ~ 13 p.i. to a similar extent (Fig. 1a and Table 1). However, disease incidence, peak score, and cumulative scores were reduced in Mdivi-1-treated compared with DMSO-treated mice (Table 1). Mdivi-1-treated mice also presented lower demyelination evaluated by Luxol Fast Blue staining and MBP immunostaining compared with DMSO-treated mice (Fig. 1b). These results show that, although Mdivi-1 does not influence the onset of EAE, overall demyelination and clinical development were reduced in treated mice compared with controls.

**Table 1:** Clinical evaluation of mice with EAE

Group	Disease onset	Disease incidence (%)	Peak score	Cumulative disease score
DMSO (*n* = 15)	13.1 ± 0.7	95%	3.6 ± 0.4	42.9 ± 5.5
Mdivi-1 (*n* = 15)	13.4 ± 0.6	65%*	1.8 ± 0.5*	16.4 ± 5.6**

Mdivi-1 was intraperitoneally administered at 25 mg/kg daily starting from day 3 p.i. Disease onset was defined as the first day of 2 consecutive days with a clinical score. Disease incidence was defined as the percentage of mice that displayed any clinical signs of disease. Cumulative disease scores were calculated as the sum of all daily scores of each individual mouse divided by the number of mice in each group. *P < 0.05, **P < 0.01, DMSO vs. Mdivi-1-treated mice

### Mdivi-1 reduced cellular infiltrates in the spinal cord of EAE mice

**Fig. 2:**
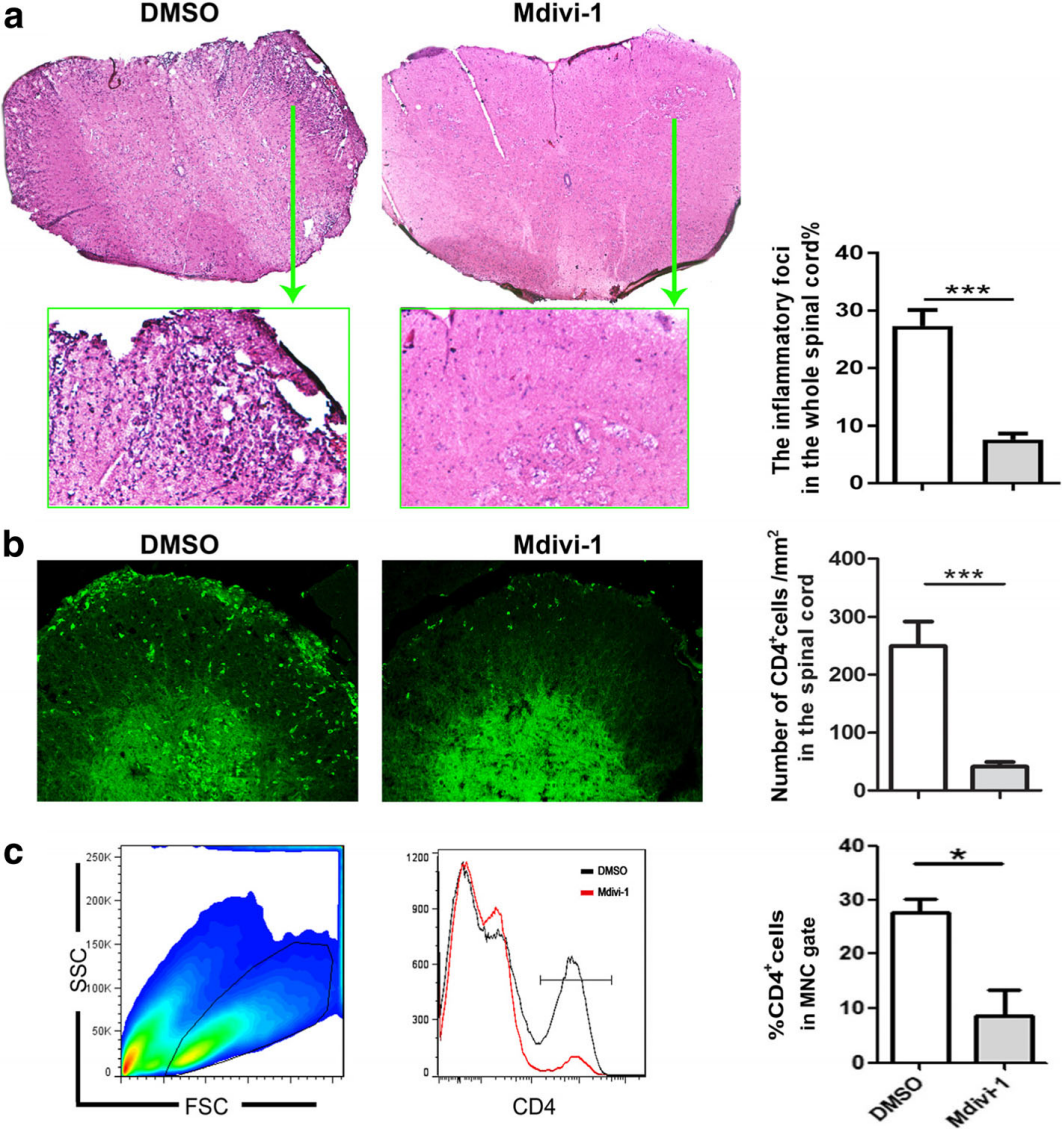
Mdivi-1 suppressed spinal cord inflammation. Lumbar regions of spinal cords were harvested from mice described in Fig. 1a for hematoxylin and eosin (H&E) staining and immunostaining of CD4 on day 28 p.i. **a** Representative microphotographs and quantitative analysis for H&E staining; **b** infiltration of CD4^+^ T cells in spinal cords (green) and quantitative analysis of spinal cords; and **c** mononuclear cells (MNCs) were isolated from the brain and spinal cord on day 28 p.i., then analyzed with flow cytometry. Mononuclear cells were gated and percentages of CD4^+^ T cells in that gate were quantitatively analyzed. Data represent mean ± SEM (*n* = 5–8 each group). *P < 0.05; ***P < 0.001. One representative of two experiments is shown

To investigate if the amelioration of EAE observed in Mdivi-1-treated mice correlated with a decrease in CNS inflammation, we analyzed the infiltration of peripheral cells in the CNS. We found that in the spinal cord of Mdivi-1-treated mice, total inflammatory infiltration foci were notably decreased compared to DMSO-treated mice, as shown by H&E staining (Fig. 2a). We then analyzed the proportion of CD4^+^ T cells by immunostaining and flow cytometry. Results showed that CD4^+^ T cells could barely be detected in the spinal cords of EAE mice injected with Mdivi-1; a large number of CD4^+^ T cells were found in EAE mice treated with DMSO (Fig. 2b, c).

### Mdivi-1 inhibited phosphorylation of Drp1 (p-Drp1; ser616) in CD4^+^ T cells

**Fig. 3:**
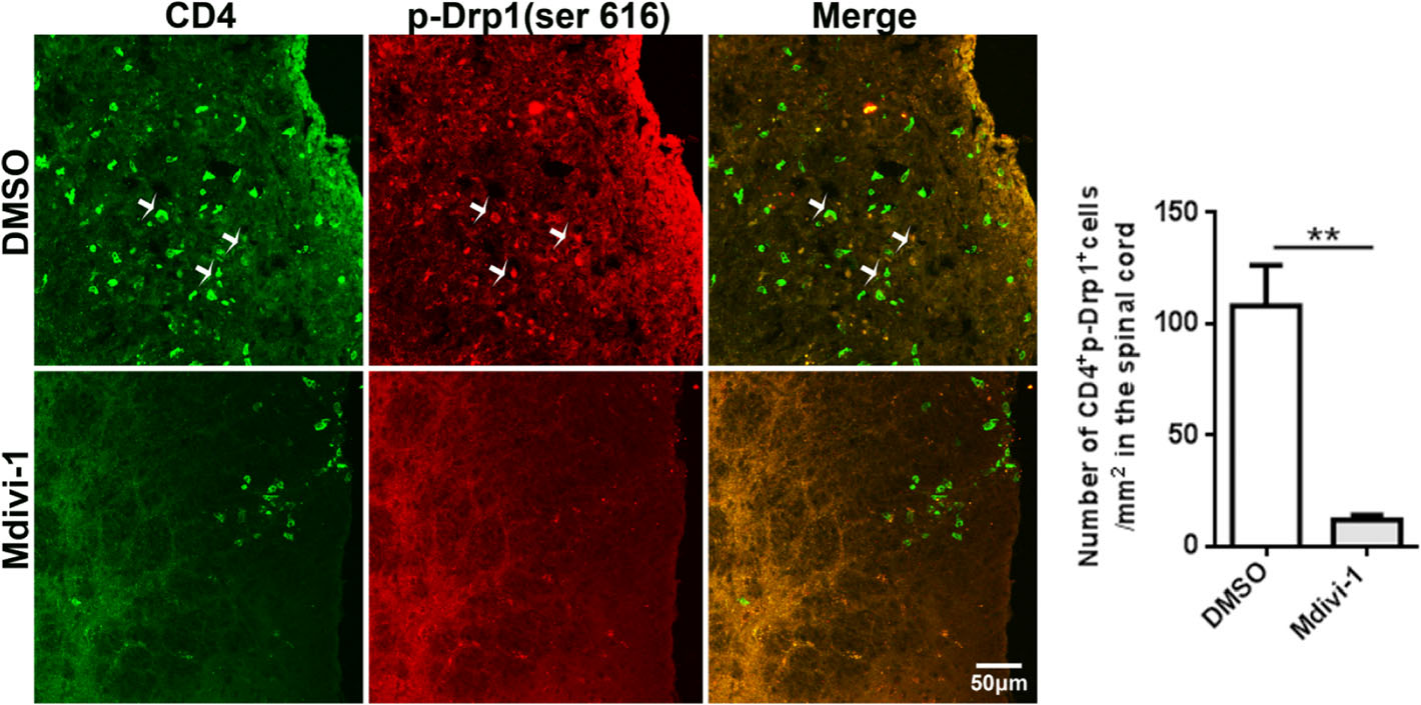
Mdivi-1 inhibited phosphorylation of Drp1 (p-Drp1; ser616) in CD4^+^ T cells. Lumbar regions of spinal cords were harvested for immunostaining of CD4, p-Drp1 (ser616) on day 28 p.i. Representative microphotographs for CD4^+^ser616^+^ cells in spinal cords and quantitative analysis of area (polygon) of CD4^+^ser616^+^ cells in spinal cords. Data represent mean ± SEM (*n* = 5–8 each group). **P < 0.001. One representative of two experiments is shown

Given that Mdivi-1 prevents Drp1 phosphorylation [26], we determined its effect on Drp1 phosphorylation in T cells by co-immunostaining of ser616 with CD4 in spinal cord tissues of EAE mice. Our results showed high number of ser616^+^CD4^+^ T cells in spinal cord of DMSO-treated control EAE mice, and its level was markedly decreased in Mdivi-1-treated mice (Fig. 3).

### Mdivi-1 inhibited Th1 and Th17 cells but upregulated IL-10^+^ T cells and Treg cells in the CNS

**Fig. 4:**
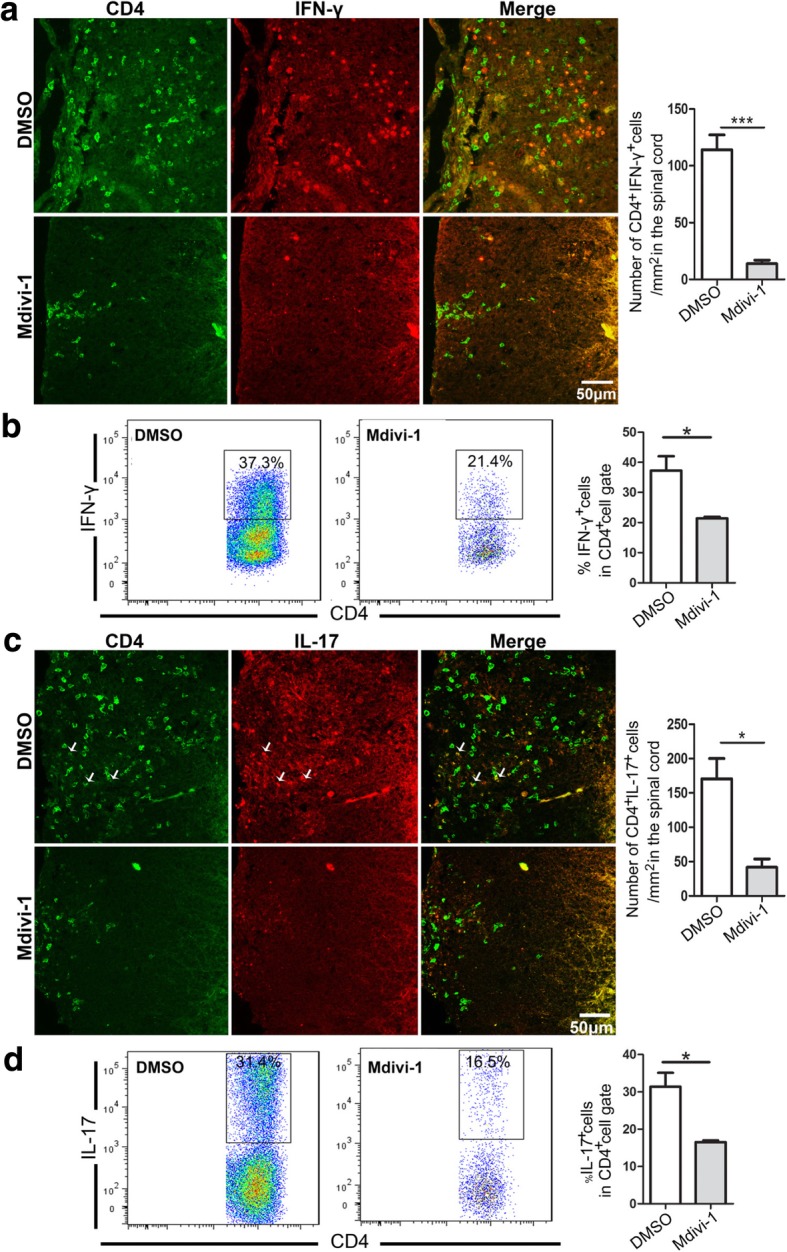
Mdivi-1 reduced the number and percentage of Th1 and Th17 cells in the CNS. Lumbar regions of spinal cords were harvested from mice described in Fig. 1a. **a** Representative microphotographs for infiltration of CD4^+^ IFN-γ^+^ cells in spinal cords and quantitative analysis of number of CD4^+^ IFN-γ^+^ cells in spinal cords. **b** Flow cytometry analysis of CD4^+^ IFN-γ^+^ cells in brain and spinal cords and quantitative analysis of percentage of IFN-γ^+^ cells in gated CD4^+^ cells described in Fig. 2c. **c** Representative microphotographs for infiltration of CD4^+^IL-17^+^ cells in spinal cords and quantitative analysis of number of IL-17^+^ cells in spinal cords. **d** Flow cytometry analysis of CD4^+^ IL-17^+^ cells in brain and spinal cords and quantitative analysis of percentage of IL-17^+^ cells in gated CD4^+^ cells described in Fig. 2c. Data represent mean ± SEM (*n* = 5–8 each group). *P < 0.05, ***P < 0.001. One representative of two experiments is shown

**Fig. 5:**
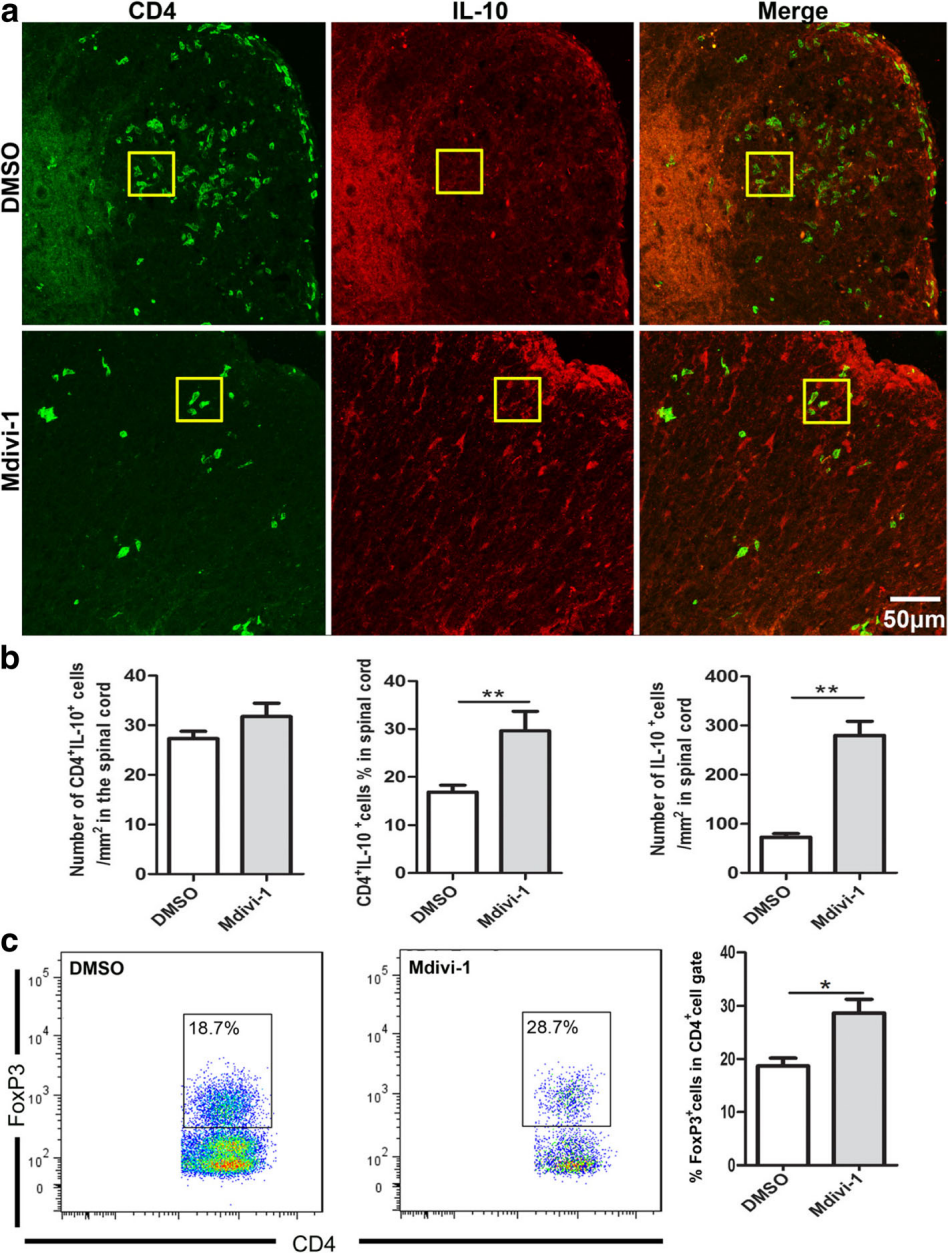
Mdivi-1 increases Treg cell number in the CNS. Lumbar regions of spinal cords were harvested from mice described in Fig. 1a. **a** Representative microphotographs for infiltration of CD4^+^ IL-10^+^ cells in spinal cords and absolute and/or relative quantitative analysis of number of CD4^+^ IL-10^+^ and CD4^−^ IL-10^+^ cells in spinal cord slices. **b** Flow cytometry analysis of CD4^+^Foxp3^+^ cells in brain and spinal cords and quantitative analysis of percentage of Foxp3^+^ cells in gated CD4^+^ cells described in Fig. 2c. Data represent mean ± SEM (*n* = 5–8 each group). *P < 0.05; **P < 0.005. One representative of two experiments is shown

Based on microenvironmental cues, naive T cells differentiate into Th1 and Th17 cells that are fundamental to EAE development [22]. To test if Mdivi-1 influences these proinflammatory T cell subsets in the CNS, we analyzed the frequency of IFN-γ^+^, IL-17^+^, and CD4^+^ T cells in Mdivi-1-treated mice and compared it with that in DMSO-treated mice. Our results showed that Mdivi-1 treatment reduced both absolute and relative numbers of IFN-γ-producing Th1 cells and IL-17-producing Th17 cells compared with DMSO-treated mice (Fig. 4a–d). We then investigated whether the mechanism of Mdivi-1 action included favoring Treg cells and IL-10 production over Th1/Th17 cells in the CNS, and found that the proportion of Treg cells and IL-10^+^CD4^+^ cells was significantly increased in the brain and spinal cords of Mdivi-1-treated mice compared with DMSO-treated mice (Fig. 5a–c).

### Mdivi-1 treatment inhibited Th1/Th17 cells and induced Treg cells in the periphery

**Fig. 6:**
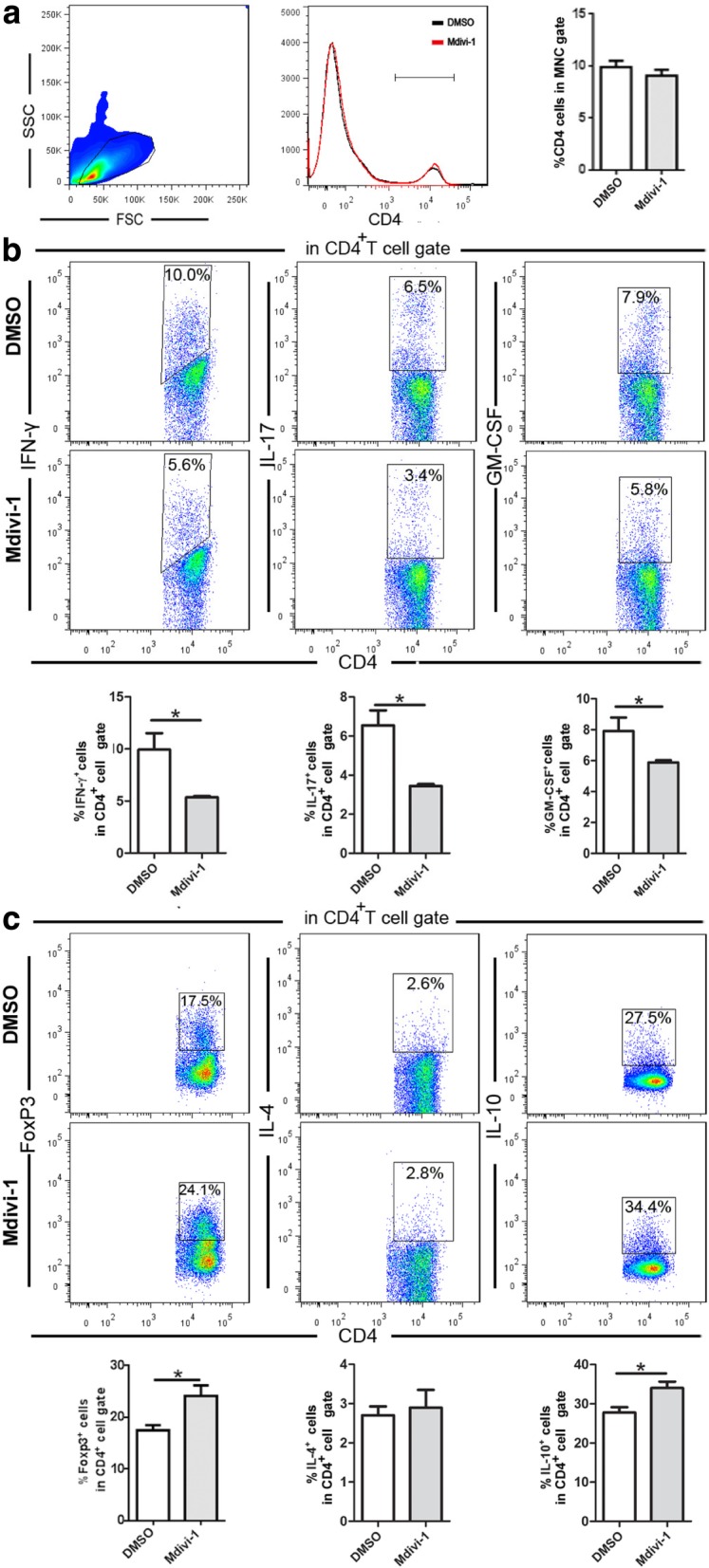
Mdivi-1 modulates systemic immune responses in EAE. Splenocytes of mice described in Fig. 1a were isolated on day 28 p.i., then directly analyzed with flow cytometry. **a** Mononuclear cells were gated and percentages of CD4^+^ T cells in that gate were quantitatively analyzed. **b** Percentage of Th1 cells in gated CD4^+^ T cells (CD4^+^ IFN-γ^+^), Th17 cells (CD4^+^ IL-17^+^), **c** Th2 cells (CD4^+^ IL-4^+^), Treg cells (CD4^+^IL-10^+^, CD4^+^Foxp3^+^). Data represent mean ± SEM (*n* = 5–8 each group). *P < 0.05. One representative of two experiments is shown

We explored whether Mdivi-1 could modulate T cells in the periphery. We found that the percentages of CD4^+^ T cells were similar in the splenocytes of two groups of mice and the percentages of IFN-γ^+^ IL-17^+^, and GM-CSF^+^ cells in gated CD4^+^ T cells (Fig. 6a) were markedly decreased in Mdivi-1-treated mice compared with DMSO-treated mice (Fig. 6b). Conversely, frequencies of CD4^+^Foxp3^+^ Treg cells and CD4^+^IL-10^+^ T cells were significantly higher in Mdivi-1-treated mice than in those of controls (Fig. 6c). Of note is that the frequency of IL-10-producing cells is higher than Treg (CD4^+^Foxp3^+^) in both Mdivi-1- and DMSO-treated mice. These Foxp3^−^ IL-10-producing T cells (6–7%) are most likely type 1 regulatory T cells (Tr1) [7]. This population, although relatively small, will be further investigated in future studies.

### Mdivi-1 suppressed auto-antigen-induced Th1/Th17 responses

**Fig. 7:**
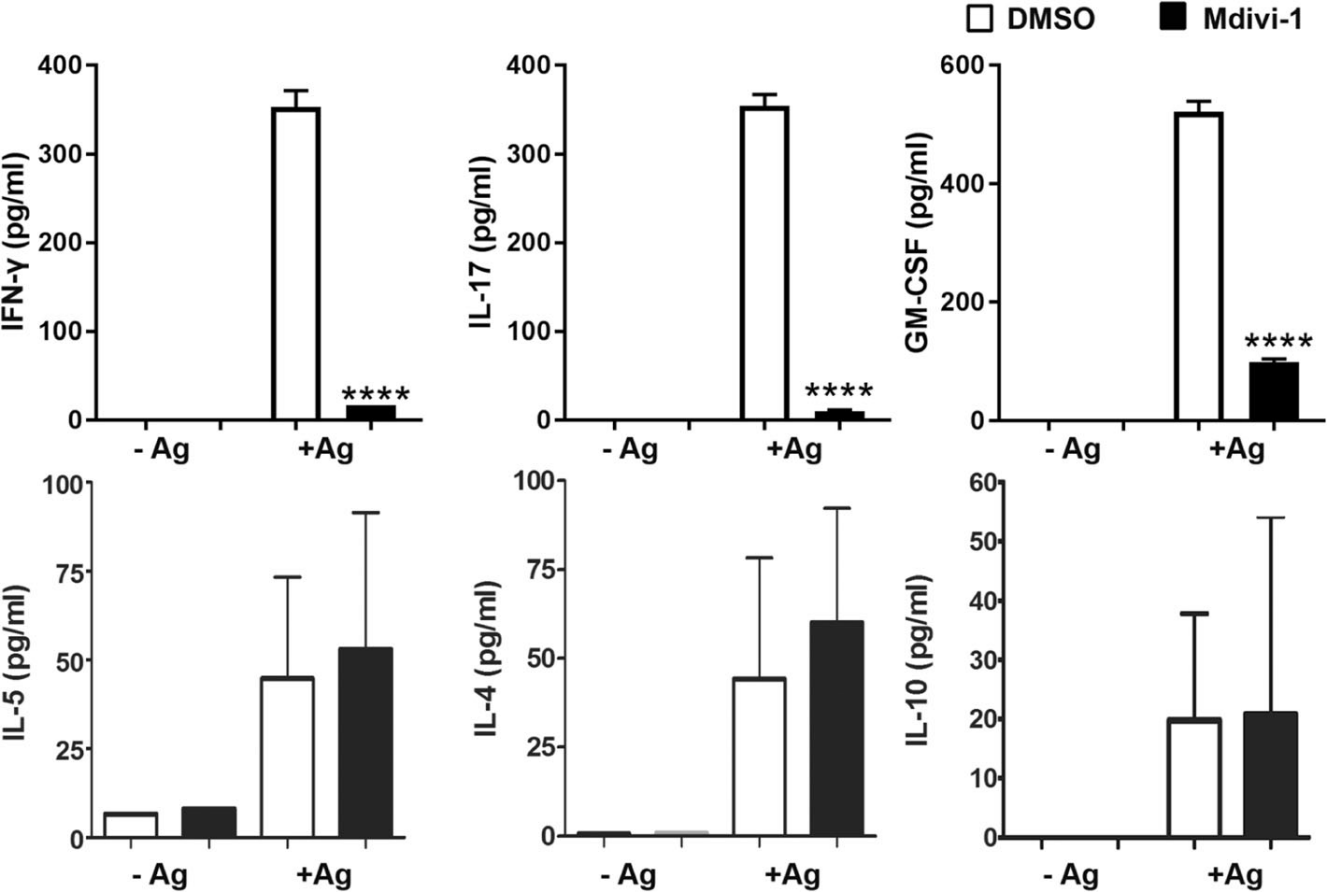
Splenocytes from Mdivi-1 treated mice did not respond to MOG35–55. Splenocytes of mice described in Fig. 1a were isolated on day 28 p.i. and cultured in complete IMDM with or without Ag (50 μg/mL MOG_35–55_) for 72 h. After the incubation period, supernatants were collected and assayed for cytokine production using ELISA kits. Data represent mean ± SEM (*n* = 5–8 each group). ***P < 0.001. One representative of two experiments is shown

Next, we analyzed whether the autoantigen-induced immune response was modulated by Mdivi-1. Splenocytes from Mdivi-1- and DMSO-treated mice were cultivated with or without MOG_35–55_ for 72 h and the supernatants were assayed for production of IFN-γ, IL-17, GM-CSF, IL-5, IL-4, and IL-10. Our results showed that cells from Mdivi-1-treated mice produced significantly lower amounts of IFN-γ, IL-17, and GM-CSF; however, production of IL-4, IL-5, and IL-10 was not significantly changed compared with that from the DMSO-treated group (Fig. 7).

## Discussion

Our present study for the first time provides evidence that treatment with Mdivi-1, a small molecule inhibitor of Drp1, significantly reduced EAE clinical severity by suppressing inflammation and demyelination in the CNS. Mdivi-1 inhibited the level of p-Drp1 (ser 616) in CD4^+^ T cells, inhibited Th1 and Th17 T cells but upregulated Foxp3^+^ Tregs in the CNS and periphery of EAE mice.

The proper functioning of mitochondria in T cells is critical for their activation, differentiation, and function [19]. T cell plasticity is influenced by their metabolism and ATP demand, where mitochondrial morphology plays an important role [14]. Recent research has shown cellular metabolism critically influences T cell differentiation and function, and emerges as a key target for therapy in the treatment of autoimmune diseases, infections, and cancer [19]. The different T cell subsets require distinct metabolic programming. For example, effector T cells have high energetic demands and require aerobic glycolysis [30], and the TCA cycle is also necessary for effector T cell function [31]. Conversely, memory and naïve T cells consume less energy and rely on fatty acid oxidation and oxidative phosphorylation [30]. Likewise, increased glycolysis and mitochondrial production of reactive oxygen species (ROS) also boost T cell calcium signaling [8] and specific effector functions, including expression of pivotal cytokines in activated CD4 T cells [9, 21]. Interestingly, although effector T cells rely on glycolysis and the TCA cycle for their metabolic supply, Treg cells require fatty acid oxidation to meet their metabolic needs [19]. The regulation of T cell differentiation and function by metabolism is closely linked to the role of the mTOR signaling pathway. mTOR is directly involved in fatty acid synthesis, promotion of aerobic glycolysis, and mitochondrial biogenesis and thus is very active in effector T cells, whereas blockage of mTOR facilitates the development of Treg cells [29]. In addition, the metabolism of amino acids, glutamine, for example, induces Th17 but limits Th1 and CTL effector cell differentiation [9].

Our results show that Mdivi-1 reduced the number of inflammatory cells while increasing the number of Treg cells in the CNS of EAE mice. How Mdivi-1 affects Th cell differentiation in EAE is unknown. Given that mitochondrial dynamics are associated with mitochondrial metabolism, bioenergetic demand, and cellular physiology [4, 6, 18], it would be reasonable to expect that mitochondrial morphology and the metabolic pathway regulate T cell subset differentiation and function [4]. Drp1, a main component of the mitochondrial fission machinery, is a major player in mitochondrial morphology and metabolism. Drp1 inhibition has been observed in T memory cells (T_M_) where mitochondria are fused together and have an elongated form; conversely, effector T cells (T_E_) have fragmented mitochondria due to an upregulation of Drp1 phosphorylation [14]. Further, Drp1-dependent mitochondrial fission correlates with an increase in ROS production, CD95L-dependent, activation-induced cell death (ACID), and cytokine production in T cells, and inhibition of Drp1 in PHA- or anti-CD3-stimulated T cells results in reduced ROS, ACID, and cytokine production [24]. Absence or diminished phosphorylation of Drp1 depolarizes mitochondria and reduces the threshold for T cell activation [2]. During T cell/APC cognate interactions, Drp1 controls T cell activation through modulating mitochondria positioning at the immune synapse [2]. Mdivi-1, through blockade of Drp1, impairs mitochondrial redistribution, which results in failure of T cell immune synapses to organize. Mdivi-1 promotes the differentiation of memory-like T cells from T_E_ cells through enhancing mitochondrial fusion [4]. In our study, we observed increased Drp1 phosphorylation in CD4^+^ T cells in EAE, an animal model of human MS, and this phosphorylation was significantly inhibited after Mdivi-1 treatment. These results provide evidence that pDrp1 inhibition may be one of the mechanisms underlying the effect of Mdivi-1 in inhibiting Th1/Th17 cells but stimulated Treg cells in EAE mice. Of note is that splenocytes of Mdivi-1-treated mice produced significantly lower amounts of IFN-γ, IL-17, and GM-CSF upon MOG_35–55_ peptide stimulation, but production of IL-4, IL-5, and IL-10 was not significantly changed. These results suggest that inhibiting myelin-reactive proinflammatory immune responses, but not inducing immunomodulatory cytokine production in the periphery, could be a major mechanism underlying the effect of Mdivi-1 treatment in EAE. Further, while IL-10 production in splenocytes remained similar, Mdivi-1 treatment significantly upregulated the percentage of FoxP3^+^CD4^+^ Tregs. These results indicate an IL-10-independent Treg function, which relies primarily on contact with target cells [7].

Although Mdivi-1 is widely considered to be a Drp1 inhibitor, it may also act via multiple mechanisms independent of Drp1 inhibition. Indeed, Mdivi-1 attenuates mitochondrial ROS production through inhibiting mitochondrial complex I, without impairing Drp1 activity or lengthening mitochondria [3]. Mdivi-1 protects against neuronal injury independently of Drp1, however, through modulating mitochondrial function and intracellular Ca^2+^ signaling [25]. Mdivi-1 also protects the BBB from leakage by decreasing its susceptibility to MMP-9 and by the degradation of claudin-5, occludin, and ZO-1 [34]. Therefore, further study to explore any pDrp1 inhibition-independent mechanisms to reprogram T cell activation, differentiation, and function, as well as BBB function, in autoimmune diseases is warranted.

## Conclusion

Our results demonstrate that Mdivi-1 has therapeutic potential in EAE by modulating the balance between Th1/Th17 and regulatory T cells. These effects, combined with the direct neuroprotective effects of Drp1 inhibition [16], as well as the capacity of Mdivi-1 to cross the BBB [5, 12], indicate that Mdivi-1 may have significant potential as a novel MS therapy in the future.

## Data Availability

The datasets used and/or analyzed during the current study are available from the corresponding author on reasonable request.

## Abbreviations

BBB: Blood-brain barrier
CNS: Central nervous system
Drp1: Dynamin-related protein 1
EAE: Experimental autoimmune encephalomyelitis
H&E: Hematoxylin and eosin
LFB: Luxol Fast Blue
Mdivi-1: Mitochondrial fission inhibitor
MNCs: Mononuclear cells
MOG_35–55_: Myelin oligodendrocyte glycoprotein peptide 35–55
MS: Multiple sclerosis
P.i.: Post immunization
T_E_: Effector T cells
T_M_: T memory cells
Tr1: Type 1 regulatory T cells
